# A phase II study of lenalidomide in patients with relapsed or refractory ATLL

**DOI:** 10.1186/1742-4690-11-S1-P4

**Published:** 2014-01-07

**Authors:** Adrienne A Phillips, Jalanni Giddings, Steven M Horwitz

**Affiliations:** 1 Division of Medical Oncology, Department of Medicine, Herbert Irving Cancer Center, New York, NY, USA; 2 Herbert Irving Cancer Center, New York, NY, USA; 3 Department of Medicine, Memorial Sloan Kettering Cancer Center, New York, NY, USA

## 

The acute and lymphomatous subtypes of ATLL have a median survival of only 6 to 13 months and there is no standard of care for relapsed or refractory disease in the US. Novel therapies are needed and we conducted a phase II study to establish the efficacy and safety of lenalidomide in ATLL. The overall response rate and safety of lenalidomide in relapsed or refractory ATLL was assessed and toxicity was classified using the NCI CTCAE. At the time of this analysis, 4 patients were enrolled from February 2011 to June 2012. The median age was 54 (range 36-56), there was 1 male and 3 females, and all patients were of Caribbean ancestry. Three patients had the acute subtype of disease and 1 had lymphomatous disease and the median ECOG performance status was 1 (0-2). The median number of prior therapies was 4 (range 2-9). Of the 4 patients enrolled, one progressed before receiving therapy and the remaining three patients received lenalidomide for one day, 15 days and 21 days respectively. There was no response seen in two patients evaluable for response after a 28 day cycle. There was no grade 3 or 4 toxicity observed and the most common toxicities were grade 1 fatigue (3/4), and grade 1 thrombocytopenia (2/4). In this small single institution experience, lenalidomide showed limited clinical activity and manageable toxicity in two evaluable patients with relapsed or refractory ATLL. Two additional patients progressed shortly after enrolling on the study.

